# Comparison of the Effects of Intravenous Fentanyl and Intravenous Dexmedetomidine on Characteristics of Spinal Anesthesia

**DOI:** 10.7759/cureus.72263

**Published:** 2024-10-24

**Authors:** Aparna Bagle, Runjhun Jain

**Affiliations:** 1 Anesthesiology, Dr. DY Patil Medical College, Hospital, and Research Centre, Dr. DY Patil Vidyapeeth (Deemed to be University), Pune, IND

**Keywords:** intravenous dexmedetomidine, intravenous fentanyl, motor block, rescue analgesia, sedation, sensory block, spinal anesthesia (sa)

## Abstract

Introduction and aim

Spinal anesthesia is a widely used technique for lower abdominal and lower limb surgeries, offering effective pain control and muscle relaxation. Various adjuvants have been explored to enhance the quality and duration of spinal anesthesia, with opioids and α-2 agonists being popular choices. Fentanyl, a potent opioid, and dexmedetomidine, a highly selective α-2 agonist, have both shown promising results when used as adjuvants. This study aimed to compare the effects of intravenous fentanyl versus intravenous dexmedetomidine on the characteristics of spinal anesthesia, including onset and duration of sensory block, hemodynamic stability, postoperative analgesia, and side effects.

Methods

This is a prospective, randomized, double-blind, comparative study involving sixty patients aged 18 to 65 years. The patients were classified as American Society of Anesthesiologists physical status I and II and were scheduled for elective infraumbilical surgery under subarachnoid block. The patients were randomly divided into two groups as follows: group D and group F. Patients of group D received IV dexmedetomidine 0.5 µg/kg and group F received IV fentanyl 1 µg/kg as premedication 5 minutes before spinal anesthesia over 10 minutes. Vital parameters, the onset of sensory and motor block, the highest level of sensory blockade achieved, regression time of spinal anesthesia by two segments, Ramsay sedation score, postoperative numerical rating scale, and time of requirement of first dose of postoperative rescue analgesic were recorded and analyzed.

Results

Both group D and group F were comparable in terms of age, gender distribution, BMI, American Society of Anesthesiologists (ASA) grading, and type of surgery. The time to achieve T10 sensory blockade was significantly faster in group F (5.5±1.27 minutes) compared to group D (6.5±1.6 minutes, p=0.01). However, the difference between the highest level of sensory blockade achieved and the time to achieve motor blockade was not statistically significant. Group D showed a significantly longer time to two-segment regression of spinal level (141.8±23.5 minutes vs. 94.33±13.6 minutes, p<0.001). Hemodynamic parameters were comparable between groups. Group D demonstrated higher Ramsay sedation scores from 10 minutes to 45 minutes postanesthesia with maximum difference at 15 minutes (p<0.001) and lower pain scores at 4 and 6 hours postsurgery (p=0.02 and p=0.008, respectively). The time to rescue analgesia was significantly longer in group D (6.9±1.5 hours vs. 5.5±0.63 hours, p<0.001). Side effects were minimal and comparable between the two groups.

Conclusion

While both fentanyl and dexmedetomidine are valuable intravenous adjuncts to spinal anesthesia, dexmedetomidine offers advantages in terms of prolonged sensory block, better postoperative analgesia, and longer time to rescue analgesia. Fentanyl, on the other hand, provides a faster onset of sensory block. Dexmedetomidine produced a slightly higher level of sedation, particularly in the early postanesthesia period. The choice between these two drugs should be tailored to the specific requirements of the surgical procedure and individual patient factors.

## Introduction

Spinal anesthesia is a widely used regional anesthesia technique, especially for surgeries below the umbilicus, such as those involving the lower abdomen, pelvis, and lower limbs [1]. It offers several benefits, including ease of administration, rapid onset, preservation of spontaneous respiration, effective motor and sensory blockade, cost-effectiveness, and safety for patients with a full stomach.

To enhance the efficacy and duration of spinal anesthesia and to reduce patient anxiety, various adjuvants are used either intravenously or intrathecally. Two common adjuvants are fentanyl, a potent synthetic opioid, and dexmedetomidine, a selective alpha-2 adrenergic agonist [2,3]. While numerous studies have explored the effects of these adjuvants when administered intrathecally, limited data exists on the comparison of intravenous fentanyl and dexmedetomidine in terms of spinal block duration, analgesic requirements, and sedation [4,5].

We aimed to compare the effects of intravenous fentanyl and dexmedetomidine on the characteristics of spinal anesthesia. Our primary objective was to assess and compare how these two agents influence the regression of spinal anesthesia by two segments (duration of sensory block) and the time to first rescue analgesia (duration of analgesia). Our secondary objective was to compare the time to achieve sensory blockade at T10, the time to achieve complete motor block, the highest level of sensory blockade achieved, intraoperative hemodynamic parameters, and the Ramsay sedation score between the two groups. This study aimed to provide insights into their relative advantages and disadvantages, ultimately enhancing patient outcomes.

## Materials and methods

This prospective, randomized, double-blind, comparative study received ethics approval (#I.E.S.C./416/2022) and CTRI clearance (#CTRI/2023/10/059118). The sample size was calculated using OpenEpi version 3 software. A confidence interval of 95% and power of study 80% were used to calculate the sample size. The difference between the onset of the sensory block between dexmedetomidine and midazolam group was 2.52±0.32 minutes and 2.97±0.64 minutes in the previous study by Kumar et al. [6]. Using the above values and OpenEpi software, the minimum sample size was 40, 20 in each group. So considering exclusion, and dropouts, we took a total sample size of 60, with 30 in each group. Patients aged 18 to 65 years, classified as American Society of Anesthesiologists physical status I and II, who were scheduled to undergo infraumbilical surgery, were included in this study. Exclusion criteria included major systemic diseases, contraindications to spinal anesthesia, known allergies to study drugs, and refusal to participate.

Pre-anesthetic checkups included history, clinical examination, and routine investigations (complete blood count, serum electrolytes, renal function test, liver function test, prothrombin time, international normalized ratio, blood glucose, blood grouping, and viral marker screening test for HIV, HBsAg, and anti-HCV). Written informed consent was obtained from all participants. Patients fasted from midnight before surgery. They were educated on using the numerical rating scale (NRS) for pain assessment.

In the operating room, standard monitors for monitoring of electrocardiogram, non-invasive blood pressure, heart rate, and peripheral oxygen saturation were attached, and baseline vital signs were recorded. A 20G IV cannula was placed, and a 500 mL loading dose of Ringer's lactate was given. Patients were randomly assigned into two groups using a computer-generated random number table. Group D received IV dexmedetomidine at a dose of 0.5 mcg/kg, and group F received IV fentanyl at a dose of 1 mcg/kg, both administered as premedication over 10 minutes. An independent anesthesiologist prepared the study drugs by premixing them to a total volume of 10 mL in identical 10 mL syringes for blinding. The attending anesthesiologist and the patients were not aware of the group allocation. Five minutes later, spinal anesthesia was given in the left lateral position using 3 mL of 0.5% bupivacaine (heavy) at the L3-L4 intervertebral space. Oxygen at 4 L/min was administered via a facemask to all patients during the procedure.

Vital parameters of heart rate (HR), mean arterial blood pressure (MAP), and oxygen saturation (SPO_2_) along with Ramsay sedation score (RSS) were recorded at baseline, after study drug administration, postdural puncture, and at intervals of 5 minutes, 10 minutes, 15 minutes, 30 minutes, 45 minutes, 60 minutes, 90 minutes, 2 hours, 4 hours, 8 hours, 12 hours, and 24 hours after spinal anesthesia (appendix 1). The sensory block was assessed using a pinprick test every 30 seconds till T10 level was achieved, noting the time to T10 level and the highest level achieved. The recovery time for sensory blockade, defined as the regression of anesthesia by two dermatomes from the maximum level, was recorded. The time to achieve motor block was evaluated using the modified Bromage scale (appendix 2). The numerical rating scale (NRS) was used to evaluate postoperative pain at 2-hour, 4-hour, 6-hour, 8-hour, 12-hour, and 24-hour intervals following the administration of spinal anesthesia. Additionally, the time to the first rescue analgesia was documented. Intravenous diclofenac sodium 75 mg in 100 mL normal saline was administered as rescue analgesia when the NRS pain score exceeded 3 or at the patient’s request. Side effects such as bradycardia (HR <50 beats/minute), hypotension (decrease in MAP below 20% of baseline or systolic blood pressure (SBP) <90 mmHg), nausea, vomiting, and rash were recorded and managed appropriately.

Data analysis was conducted using SPSS version 20 (Chicago, IL: SPSS Inc.). The independent Student's t-test was used to analyze the continuous variables, whereas the chi-square test was used to analyze the categorical variables. A p-value of less than 0.05 was considered statistically significant.

## Results

**Figure 1:**
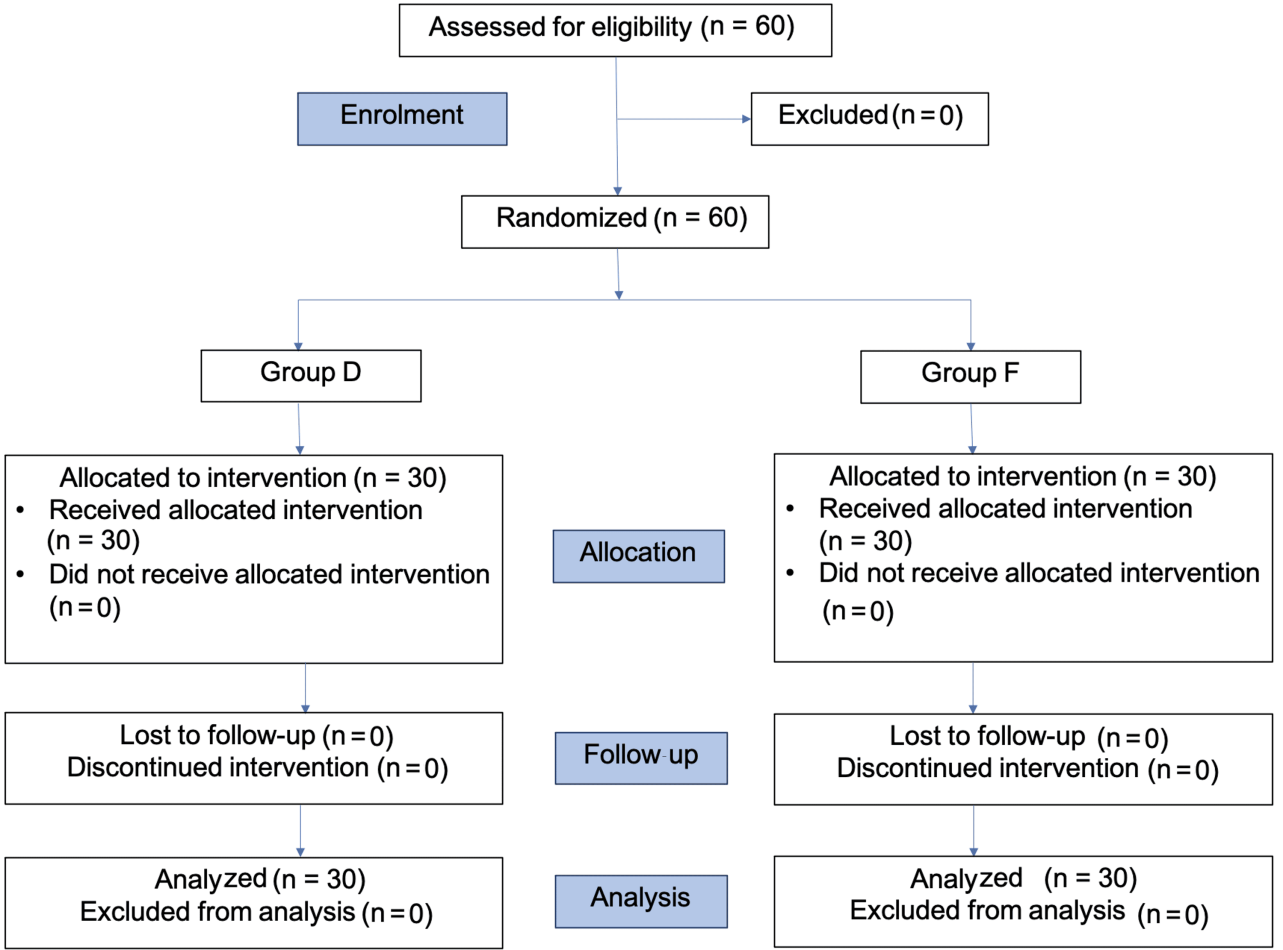
Consolidated Standards of Reporting Trials (CONSORT) flow diagram. Group D: dexmedetomidine group; group F: fentanyl group

Sixty patients were enrolled and randomly allocated into two groups of 30 each (Figure 1). The demographic data and the duration of surgery in both groups were comparable (Table 1). 

**Table 1:** Demographic data and duration of surgery. Group D: dexmedetomidine group; group F: fentanyl group

Parameter	Group D	Group F	p-Value
Age in years (mean±SD)	41.2±12.3	41.5±12.4	0.909
Gender			0.739
Male	25 (83.3%)	24 (80%)	
Female	5 (16.7%)	6 (20%)	
BMI in kg/m^2 ^(mean±SD)	22.68±2.10	22.41±1.46	0.581
Duration of surgery in min (mean±SD)	60.3±22.9	60.6±15.1	0.94

Table 2 depicts a comparison of the type of surgery between the two groups. A majority of the patients from Group D underwent lower limb implant removal (16.7%) and fistulectomy (16.7%) and most of the patients from Group F underwent fistulectomy (16.7%). The difference in the groups was not statistically significant when chi-square test was applied (p>0.05) (Table 2).

**Table 2:** Comparison of type of surgery between two groups. Group D: dexmedetomidine group; group F: fentanyl group; ORIF: open reduction and internal fixation; DJ: double J (stents)

Type of surgery	Group D	Group F	p-Value
Circumcision	2 (6.7%)	2 (6.7%)	0.27
Distal fibula fracture-ORIF with plating	1 (3.3%)	0	
DJ stent removal	4 (13.3%)	1 (3.3%)	
DJ stenting	1 (3.3%)	3 (10%)	
Fasciotomy for cellulitis	2 (6.7%)	0	
Perianal fistulectomy	4 (13.3%)	5 (16.7%)	
Haemorrhoidectomy	2 (6.7%)	4 (13.3%)	
Lower limb implant removal	5 (16.7%)	5 (16.7%)	
Inguinal hernia repair	4 (13.3%)	4 (13.3%)	
Jaboulay’s procedure	0	2 (6.7%)	
Orchidectomy	1 (3.3%)	1 (3.3%)	
Secondary suturing of laceration	1 (3.3%)	0	
Ulcer debridement	3 (10%)	4 (13.3%)	

Time to achieve T10 sensory as well as motor blockade was longer in group D compared to group F but the difference was significant only with time to achieve T10 sensory blockade when independent t-test was applied (p<0.05). The time to two-segment regression of spinal level was significantly longer in group D compared to group F (p<0.001). Group D had a significantly longer time to rescue analgesia (6.9±1.5 hours) compared to group F (5.5±0.63 h) and this difference was statistically significant (p<0.001) (Table 3).

**Table 3:** Effect on onset and regression of block. Group D: dexmedetomidine group; group F: fentanyl group

Blockade	Group D	Group F	p-Value
Mean time to achieve T10 sensory blockade (min)	6.5±1.6	5.5±1.27	0.01
Mean time to achieve motor blockade (min)	2.68±1.01	2.3±0.63	0.11
Mean time to two-segment regression of spinal level (min)	141.8±23.5	94.33±13.6	<0.001
Mean time to first request of analgesia (h)	6.9±1.5	5.5±0.63	<0.001

Table 4 depicts a comparison of the highest level of sensory blockade achieved between the two groups. The group differences were not statistically significant (p>0.05) (Table 4).

**Table 4:** Comparison of the highest level of sensory blockade achieved between the two groups. Group D: dexmedetomidine group; group F: fentanyl group

Highest level of sensory blockade	Group D	Group F	p-Value
T10	3 (10%)	6 (20%)	0.38
T8	17 (56.7%)	17 (56.7%)	
T6	8 (26.7%)	7 (23.3%)	
T4	2 (6.7%)	0	

**Figure 2:**
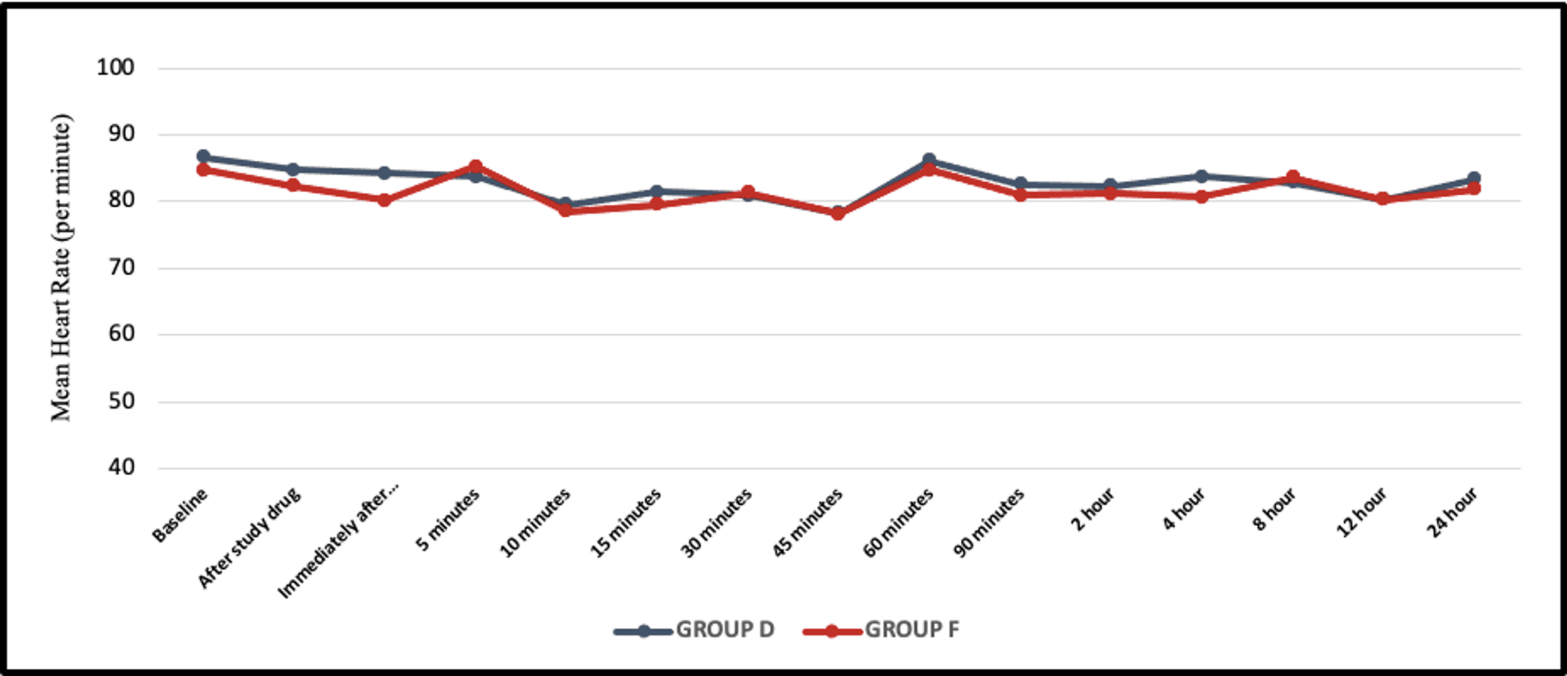
Line graph represents a comparison of mean heart rate (per minute) between the two groups. Group D: dexmedetomidine group; Group F: fentanyl group

**Figure 3:**
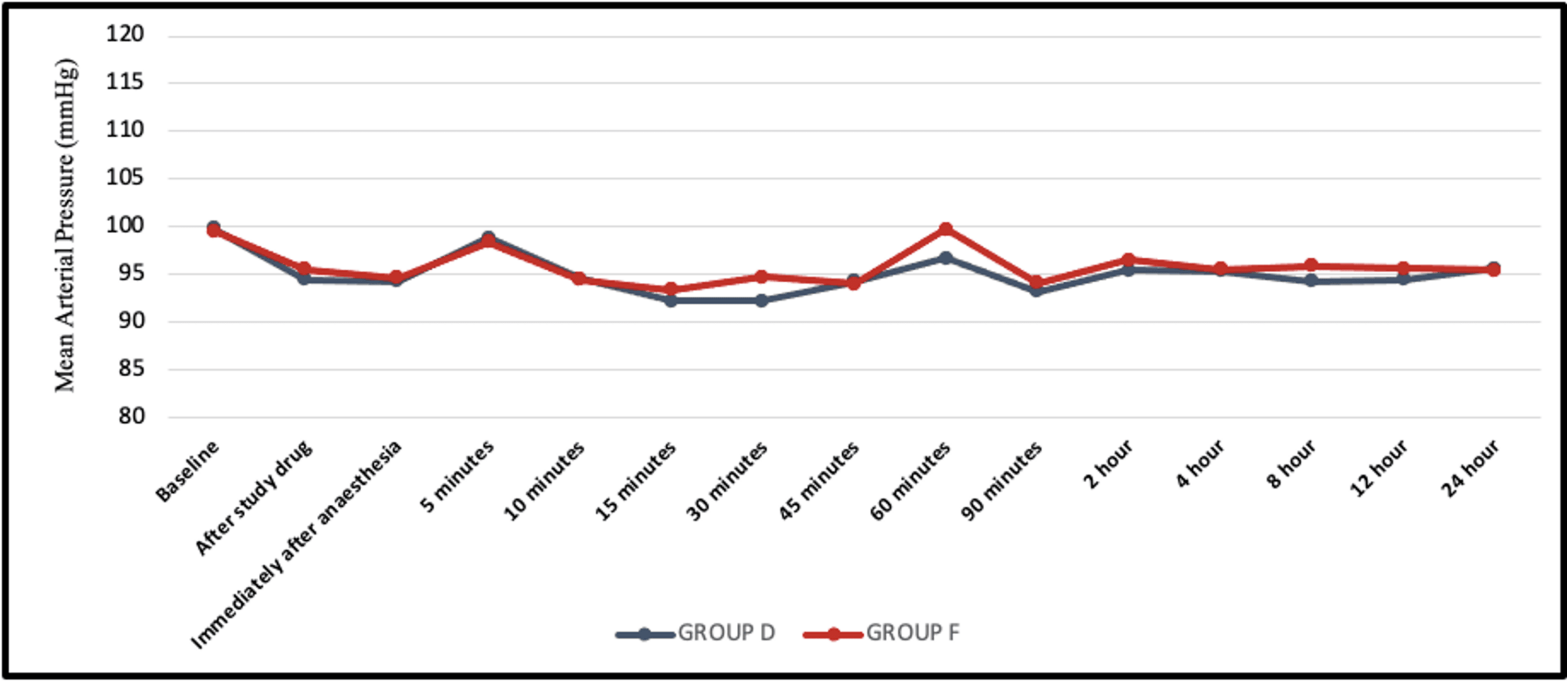
Line graph represents a comparison of mean arterial pressure (MAP) between the two groups. Group D: dexmedetomidine group; group F: fentanyl group

Patients in both groups were hemodynamically stable throughout the surgery and postoperative observation period. Mean values of HR, MAP, SpO_2_, and RR were comparable in the two groups (Figures 2, 3).

**Figure 4:**
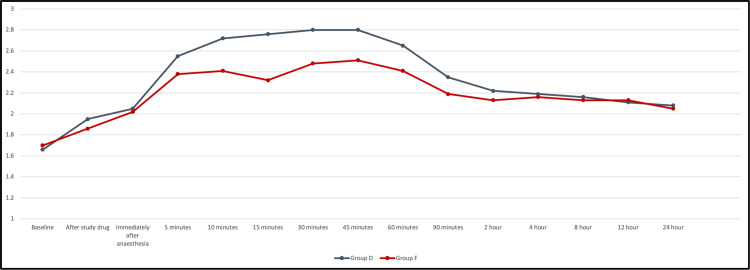
Line graph represents a comparison of Ramsay sedation score between the two groups. Group D: dexmedetomidine group; group F: fentanyl group

After the study drug administration, the RSS of group D was slightly higher than group F for most of the study time but the difference was statistically significant only from 10 minutes to 45 minutes. The maximum difference was at 15 minutes where group D showed a higher RSS score (2.76±0.4) compared to group F (2.32±0.49), with a p<0.001 (Figure 4).

**Figure 5:**
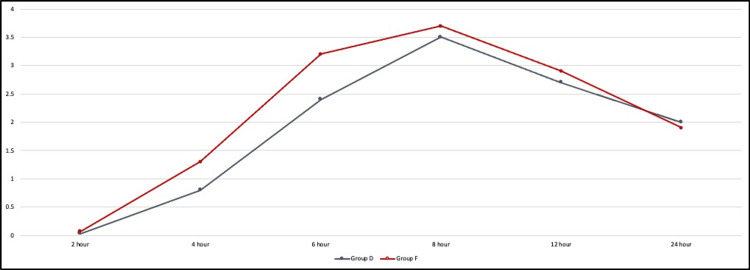
Line graph represents a comparison of numeric rating scale between the two groups. Group D: dexmedetomidine group; group F: fentanyl group

Both groups experienced a gradual increase in pain scores, peaking at 8 hours post-intervention. Significant differences were observed at 4 hours (group D: 0.8±0.88 vs. group F: 1.3±0.79, p=0.02) and 6 hours (group D: 2.4±1.2 vs. group F: 3.2±0.97, p=0.008), with group F reporting higher pain levels. By 8 hours, these differences resolved, and both groups had similar pain scores for the rest of the observation period (Figure 5).

Group D had a significantly longer time to the first rescue analgesia (6.9±1.5 hours) compared to group F (5.5±0.63 hours) and this difference was statistically significant with a p<0.001 when independent t-test was applied. There was a significant difference in time to total analgesic requirement between the two groups (intravenous diclofenac sodium 122.5±45.09 mg in group D vs. 142.5±22.88 mg in group F) with a p-value of 0.03 (Table 5).

**Table 5:** Time to the first rescue analgesia in the two groups and the total analgesic requirement in 24 hours. Group D: dexmedetomidine group; group F: fentanyl group

Variables	Group D	Group F	p-Value
Time to first rescue analgesia in h (mean±SD)	6.9±1.5	5.5±0.63	<0.001
Total analgesic consumption in 24 h in mg (mean±SD)	122.5±45.09	142.5±22.88	0.03

Side effects were minimal in both groups. One (3.3%) patient from group D had hypotension and one (3.3%) patient in group F had vomiting and the difference in the groups was not significant (p>0.05).

## Discussion

It has been proposed that the main mechanism underlying dexmedetomidine's analgesic effects is the spinal mechanism [7]. When injected intravenously, dexmedetomidine acts at both spinal and supraspinal levels to provide analgesia [8,9]. Fentanyl, a lipophilic µ-receptor agonist, interacts with opioid receptors in the spinal cord and can spread supraspinally [10]. This study compared the effects of intravenous dexmedetomidine (group D) and fentanyl (group F) on spinal anesthesia characteristics. The main findings of this study can be summarized and compared with existing literature as below.

Demographic comparison

Both groups were comparable in age, gender, and BMI (p>0.05). There was no statistical difference in the type of surgery and the mean duration of surgery between the two groups (p>0.05).

Onset of sensory and motor block comparison

The time to achieve T10 sensory blockade was significantly faster in group F (5.5±1.27 minutes) compared to group D (6.5±1.6 minutes, p=0.01). Group D took longer to reach motor blockade (2.68±1.01 minutes) compared to group F (2.3±0.63 minutes), but this was not significant (p=0.11). Previous studies, including those by Kaya et al., Verghese et al., Agrawal et al., and Abdallah et al. found that the difference in the onset times of sensory and motor blocks between intravenous dexmedetomidine and control groups was not significant [11-14]. Lee et al. also found no discernible difference in onset times [15]. However, Harsoor et al. reported faster sensory block onset with intravenous dexmedetomidine (66±44.14 s) compared to a control group (129.6±102.4 s) [16]. Reddy et al. also found that intravenous dexmedetomidine premedication resulted in a quicker sensory block onset to the T10 segment (2.91±1.16 minute) vs. clonidine (3.58±1.06 minute) [17].

Highest level of sensory blockade achieved

The difference between the highest level of sensory blockade achieved between the two groups in our study was not statistically significant. According to the study by Sanjay et al., premedication with a single dose of IV dexmedetomidine led to an increase in the maximum upper level of the sensory component of spinal anesthesia (6.42±3.21 vs. 4.8±1.21 thoracic segments with midazolam sedation) [6]. Although the exact mechanism of intravenous dexmedetomidine's effect on spinal anesthesia is yet unknown, it involves vasoconstriction, direct analgesia, and/or supraspinal effects [18]. Furthermore, dexmedetomidine selectively blocks the A α-fibers which are involved in sensory conduction over the C fibers that are involved in motor transmission, leading to a higher degree of differential blockage [14].

Two-segment regression of sensory blockade

Previous clinical trials have shown that intravenous dexmedetomidine can extend the sensory blockade duration and, to a smaller extent, the length of the motor blockade [14]. A few studies, meanwhile, have directly contrasted various doses of dexmedetomidine. In the study by Lee et al., single doses of 0.5 μg/kg and 1 μg/kg of dexmedetomidine were found to extend the two-segment regression durations of both sensory and motor blocks [15]. The difference in length of spinal anesthesia was not statistically significant between the D-1 and D-0.5 groups. Note that in a prior investigation, dexmedetomidine showed an analgesic ceiling effect at a dose of 0.5 μg/kg [19].

The time to two-segment regression of spinal level in our study was significantly longer in group D (141.8±23.5 minutes) compared to group F (94.33±13.6 minutes, p<0.001). This prolongation of the sensory block with intravenous dexmedetomidine aligns with several previous studies [9,11,14,16]. Verghese et al. reported an increased duration of sensory block in group D (120±22.2 minutes) compared to the control group (76±20.3 minutes) [12]. Niu et al. compared intravenous and intrathecal dexmedetomidine and reported an increased duration of sensory block in the intravenous group (mean difference = 73.55; 95% CI = 55.69, 91.40; p<0.00001; I^2^ = 89%) [20].

Hemodynamic parameters

No significant differences in heart rate, mean arterial blood pressure, or respiratory rate were observed between the two groups. Dexmedetomidine might produce bradycardia and hypertension if administered rapidly and hence it should be administered slowly over 10 minutes [6]. To ensure blinding, both drugs were administered intravenously over 10 minutes. The usual biphasic response seen with dexmedetomidine was not observed. This could be a result of the sympathetic blockade resulting from spinal anesthesia, the slow administration of a low dose, and sufficient hydration preoperatively.

All patients in our study received oxygen at 4 L/minute via a facemask throughout the procedure and postoperatively until two hours postspinal block. This could explain a constant SpO_2_ of 100% in both groups throughout the procedure. Even after two hours, the difference in mean SpO_2_ between the two groups was not statistically significant.

Ramsay sedation score

The Ramsay sedation score (RSS) was significantly higher in group D in comparison to group F from 10 minutes to 45 minutes post-anesthesia with a significant difference at 15 minutes (p<0.001). This increased sedation with dexmedetomidine is a well-documented effect. Niu et al. found that intravenous dexmedetomidine provided better sedation compared to intrathecal dexmedetomidine during spinal anesthesia for cesarean section [20].

Numeric rating scale

NRS scores for pain were significantly lower in group D than in group F at 4 hours (0.8±0.88 vs. 1.3±0.79, p=0.02) and 6 hours (2.4±1.2 vs. 3.2±0.97, p=0.008) post-surgery. This suggests that dexmedetomidine offers better analgesia than fentanyl, as supported by Wu et al.'s meta-analysis, which found dexmedetomidine more effective as an adjuvant to local anesthetics [21]. Kaya FN et al. also demonstrated that dexmedetomidine outperforms other intravenous adjuvants like midazolam and normal saline, with their study showing earlier onset of visual analog scale (VAS) scores reaching 4 in the midazolam and saline groups [11].

Time to first rescue analgesia

The time to rescue analgesia was significantly longer in group D (6.9±1.5 hours) compared to group F (5.5±0.63 hours, p<0.001). A study by Kubre et al. showed that a single dose of intravenous dexmedetomidine of 0.5 μg/kg prolongs spinal anesthesia with hyperbaric bupivacaine and reduces the requirement for analgesics [8]. This prolonged duration of analgesia with dexmedetomidine was also noted in a study by Gupta et al., which showed that intrathecal dexmedetomidine prolonged the time to first analgesic request (251.7±30.7 minutes) compared to fentanyl (168.96±15.96 minutes) in patients undergoing lower abdominal surgery under spinal anesthesia [5]. A systemic review and meta-analysis by Abdallah et al. concluded that dexmedetomidine prolonged the time of the first analgesic after spinal anesthesia [14]. 

Side effects

The incidence of side effects was low and similar between the two groups as follows: one case of intraoperative hypotension (drop in mean arterial pressure of >20% from baseline) in group D and one case of nausea followed by vomiting in group F. This aligns with the known safety profiles of these drugs as adjuvants to spinal anesthesia [9]. In Siddiqui et al.'s trials, seven patients in group F required rescue antiemetics, whereas none was required in group D, indicating a higher incidence of post-operative nausea and vomiting (PONV) with fentanyl [22]. PONV, a common opiate-related side effect, can be managed with a 5HT3 receptor antagonist alone or with dexamethasone. Previous studies by Subasi et al. and Bakan et al. support these findings [23,24], showing that about 50% of patients on opioids experience PONV [25]. Neither group had any other adverse effects, confirming their suitability as intravenous adjuncts to spinal anesthesia.

Limitations

There were certain limitations to our study. For instance, the study included only 60 patients (30 in each group), which may limit the generalizability of the results and the power to detect smaller differences between the groups. Only ASA I and ASA II patients were included in the study. Fixed doses of fentanyl (1 μg/kg) and dexmedetomidine (0.5 μg/kg) were used, which may not account for individual patient variability or optimal dosing for different patient types.

## Conclusions

Both fentanyl and dexmedetomidine are effective adjuvants to spinal anesthesia, each with distinct advantages. Fentanyl provides a faster onset of sensory blockade, which is useful for rapid anesthesia needs. In contrast, dexmedetomidine significantly prolongs the duration of the sensory block, offering superior postoperative pain control with lower pain scores at 4 and 6 hours and a longer time to rescue analgesia. Both drugs maintain stable hemodynamics and have a low incidence of side effects, indicating their safety. Dexmedetomidine also induces slightly higher sedation early post-anesthesia, which can be beneficial in certain scenarios. The choice between them should be tailored to the specific surgical requirements and patient needs. Further research with larger, diverse populations could help validate these findings and explore their applications in different surgical settings.

## Appendices

Appendix 1

**Table 6:** Ramsay sedation scale.

Sedation level	Score
Patient is anxious and agitated or restless, or both	1
Patient is cooperative, oriented and tranquil	2
Patient responds to commands only	3
Patient exhibits brisk response to light glabellar tap or loud auditory stimulus	4
Patient exhibits a sluggish response to light glabellar tap or loud auditory stimulus	5
Patient exhibits no response	6

Appendix 2

**Table 7:** Modified Bromage scale to assess motor power.

Grade	Definition
0	No motor block
1	Inability to raise extended leg; able to move knees and feet
2	Inability to raise extended leg and move knee; able to move feet
3	Complete block of motor limb
